# The relations between mental well-being and burnout in medical staff during the COVID-19 pandemic: A network analysis

**DOI:** 10.3389/fpubh.2022.919692

**Published:** 2022-08-10

**Authors:** Chen Chen, Fengzhan Li, Chang Liu, Kuiliang Li, Qun Yang, Lei Ren

**Affiliations:** ^1^Military Medical Psychology School, Air Force Medical University, Xi'an, China; ^2^Brain Park, Turner Institute for Brain and Mental Health and School of Psychological Sciences, Monash University, Clayton, VIC, Australia; ^3^School of Psychology, Army Medical University, Chongqing, China

**Keywords:** COVID-19, medical staff, mental well-being, burnout, network analysis

## Abstract

**Background:**

Although poor mental well-being (MW) has been documented among individuals experiencing burnout during the coronavirus-19 (COVID-19) pandemic, little is known about the complex interrelationship between different components of MW and burnout. This study investigates this relationship among medical staff during the COVID-19 pandemic through network analysis.

**Methods:**

A total of 420 medical staff were recruited for this study. Components of MW were measured by the 14-item Warwick-Edinburgh Mental Well-being Scale (WEMWBS), and components of burnout were measured by a 15-item Maslach Burnout Inventory-General Survey (MBI-GS) Questionnaire. Network structure was constructed *via* network analysis. Bridge variables were identified *via* the bridge centrality index.

**Results:**

The edges across two communities (i.e., MW community and burnout community) are almost negative, such as edge MW2 (“Useful”) – B14 (“Worthwhile”) and edge MW1 (“Optimistic about future”) – B13 (“Happy”). The edges within each community are nearly positive. In the MW community, components MW1 (“Optimistic about future”) and MW6 (“Dealing with problems”) have the lowest bridge centrality. And in the community of burnout, components B13 (“Happy”) and B14 (“Worthwhile”) have the lowest bridge expected influence.

**Conclusion:**

We present the first study to apply the network approach to model the potential pathways between distinct components of MW and burnout. Our findings suggest that promoting optimistic attitudes and problem-solving skills may help reduce burnout among medical staff during the pandemic.

## Introduction

The COVID-19 pandemic caused by the new SARS-CoV-2 virus puts tremendous pressure on the medical staff. Medical staff worldwide are facing unprecedented challenges, such as the lack of medical resources, heavy work, and the current tense epidemic situation (1). As a result, the physical health status of medical staff has become a public concern. According to previous systematic review research, as of May 8, 2020, there were no <152,888 infected cases and 1,413 deaths among medical staff worldwide (2). Furthermore, the psychological status of medical staff is not optimistic. At the early stage of the pandemic, mental health concerns (e.g., insomnia, anxiety, depression, and post-traumatic stress disorder symptoms) were frequently reported by medical staff (3–5). With the aggravation of the COVID-19 pandemic and the continuous impact on the medical and health system, medical staff inevitably have more hidden psychological problems, such as burnout (6). Burnout can be caused by several factors, including environmental factors, such as workplace conflict, increased burdens, and personal factors, such as work-life imbalance, perfectionism, or personality traits associated with the obsessive–compulsive disorder (7). During the COVID-19 pandemic, these factors were more pronounced (7). During the COVID-19 pandemic, the prevalence of burnout among medical staff has not been accurately investigated. However, according to the Medscape National Physician Burnout and Suicide Report, the burnout rate of medical staff has reached 43% (8). Maslach et al. (9) believed that burnout is characterized by emotional exhaustion, cynicism, and decreased professional efficacy of professional staff, which is also called Burnout Syndrome. Burnout has a significant effect on the medical staff, which manifests as a decrease in enthusiasm for patients (10), indifference to medical care, strained relationships with patients, heightened conflict with colleagues (11), pointless medical work, and low self-esteem (12). Considering these negative consequences, there is an urgent need to address burnout among medical staff during the pandemic (6, 13).

One candidate target to address burnout among medical staff is the positive psychological function, such as mental well-being (14). Many studies have shown that positive mental well-being may reduce burnout (15, 16). MW is defined as the positive process of recognizing and making choices for a healthy and cheering life (17). In real life, MW may reflect as positive life experiences that consist of good social support, positive emotions, and satisfaction in life and work (18). For this particular group of medical staff, MW plays an essential role in developing and maintaining medical personnel's sympathy and compassion for patients and their dedication to the strict aspects of medicine (19), both of which are crucial to alleviating burnout. Thus, it is feasible to alleviate burnout among medical staff by targeting MW from the theoretical perspective. And from a practical standpoint, the responsibility to promote nurses' MW has been written into Article 5 of the Code of Ethics for Nurses with Interpretive Statements to cope with nurses' burnout (20). In addition, some studies have also shown that some MW components (e.g., good social support) may alleviate the burnout of medical staff (21, 22).

Previous studies examined the relations between MW and burnout at a construct level (*via* sum scores of self-report measures) (23, 24). Inevitably, the utilization of sum scores ignores that MW and burnout have different components. In fact, MW is a complex psychological construct composed of psychological function, emotion, and interpersonal relationships (25). Burnout is also a heterogeneous syndrome that features distinct cognitive, emotional, and physical components (9). Therefore, ignoring the different components of MW and burnout (i.e., using total scores on self-report questionnaires) may be problematic as it may overlook the differential association between MW and burnout components and restrict the development of intervention methods. Therefore, a more fine-grained approach (i.e., examining the relationship between MW and burnout at the component level rather than the total-score level) should be adopted to move forward.

Network analysis is a novel statistical approach that models the relationships between psychological constructs at the component level. The network consists of two parts: nodes, representing variables, and edges, representing relationships between variables (26, 27). Several advantages of network analysis make it a suitable analytical technique for the current study. First, previous studies have explored the internal structures of MW and burnout, respectively, with network analysis and concluded that network analysis is a valuable tool for deepening understanding of these two psychological constructs (28, 29). Second, existing research mainly focused on examining the relationship between MW and burnout at a construct level (*via* sum scores). This may ignore the unique relationships among different components of MW and burnout (30, 31). Using network analysis may contribute to existing knowledge by elucidating the relationship between MW and burnout at a component level. Third, network analysis may reveal the relation between MW and burnout components by partial correlation and regularization process, which may effectively solve the traditional problem of over-interpretation and fail to replicate results (32). Fourth, the utilization of the bridge expected influence index (i.e., the sum of the value of all edges connecting a specific node with other community nodes) may help quantify the protective ability of different MW components on burnout from a network perspective and may provide some references for the potential intervention methods (33). In a word, network analysis could provide new theoretical viewpoints to comprehend the relations between MW and burnout at the component level.

Using network analysis, this study investigated the relationships between MW and burnout components. This study has two aims. First, to explore the relations between different components of MW and burnout. Second, using bridge expected influence to identify the most influential node within the MW-burnout network. Existing research showed that MW is negatively associated with burnout (15, 16). Hence, we hypothesized that the edges between MW and burnout of medical staff are primarily negative. Furthermore, previous studies demonstrated that the relationship between MW and the emotional exhaustion dimension of burnout is more potent than between MW and other dimensions of burnout (34). Thus, we hypothesized that the emotional exhaustion dimension of burnout may have the strongest negative interrelationship with the MW community. Third, previous studies have shown that positive emotions and good problem-solving skills may alleviate burnout (35, 36). Thus, we hypothesized that MW components reflect positive emotions and problem-solving skills may be the bridging nodes to the burnout community.

## Methods

### Ethics statement

The data collection procedure followed the Declaration of Helsinki and was approved by the Ethics Committee of the First Affiliated Hospital of the Fourth Military Medical University (Project No. KY20202063-F-2).

### Participants

Data were collected between 16 and 18 April 2021 *via* paper and pencil tests. Four hundred and fifty-eight medical staff from Xijing Hospital in Shaanxi Province of China participated in this study. All participants provided informed consent before taking part. Demographic data were collected at the beginning of the study. Thirty-eight participants were excluded due to failing the two honesty check items (e.g., The participants didn't choose the second option when asked to choose “Please choose the second option”) or demographic items (e.g., In response to the “Age” question, participants filled in 10 years old). The final sample consisted of 420 participants. Our sample error is 4.8% when the confidence interval is 95%.

### Measures

#### Components of mental well-being

The 14-item Warwick-Edinburgh Mental Well-being Scale (WEMWBS) is a short, efficient, psychological measurement scale used to measure MW (37, 38). The item is rated on a five-point Likert scale, ranging from 1 (“none of the time”) to 5 (“all of the time”). The score should be based on the simple sum of the project, and the total score was used to evaluate MW. In this research, the Chinese version of WEMWBS was used to assess the diverse components of MW (37, 38). The Chinese version of WEMWBS has good reliability and validity. The Cronbach'sα of WEMWBS in this study was 0.96.

#### Components of burnout

Maslach Burnout Inventory-General Survey (MBI-GS), which is widely used to measure occupational burnout, is jointly compiled by American social psychologists, Maslach and Jaskson (9). Scores for each item range from 0 (never) to 6 (very frequently), the total score represents different levels of burnout. Through exploratory factor analysis, Li and colleagues found that the general MBI-GS scale has an item with a high cross load in the cynicism dimension. After deleting this item, a more ideal MBI-GS (Chinese version) is obtained. This Chinese version of MBI-GS consists of 15 items covering three dimensions: emotional exhaustion (from item 1 to item 5), cynicism (from item 6 to item 9), and reduced professional efficacy (from item 10 to item 15; reverse scoring). This study adopts the MBI-GS Chinese version because the scale has more localization characteristics with good reliability and validity (39, 40). The Cronbach's α of MBI-GS in this study was 0.93.

### Data analysis

The present network was estimated *via* the Gaussian Graphical Model (GGM) (32). GGM belongs to an undirected network, and its edge represents the partial correlation between two nodes after statistical control of all other nodes in the network. As recommended by previous studies, the estimation of network structure was based on Spearman correlations to account for the ordinal nature of the present dataset (32, 41). The least absolute shrinkage and selection operator (LASSO) regularization algorithm was adopted to obtain a sparse network that reflects the true network structure. During the regularization process, edges (i.e., partial correlations between nodes after adjusting the effect of all other nodes) with small coefficients were removed, leaving the network with the most robust edges (32, 42). The tuning parameter for regularization was based on the Extended Bayesian Information Criterion (EBIC). Following the recommendation (32, 43), the tuning parameter value was set to 0.5 to balance the trade-off between sensitivity and specificity. The force-directed layout algorithm (i.e., the Fruchterman-Reingold algorithm) was adopted to generate the network layout (44). This layout algorithm lays nodes with stronger and more numerous relations more centrally in the network and weakly associated nodes on the periphery. Within the presented network, positive correlations were depicted as blue edges, while negative correlations were depicted as red edges. The magnitudes of correlations were reflected as edge thickness, with thicker edges representing stronger correlations. The aforementioned steps were carried out *via* the R-package *qgraph* (45).

To examine the interrelationships between MW components and well-being components, we manually divided nodes into two communities, namely, the MW community (items from WEMWBS) and the burnout community (items from MBI-GS). A previous study has shown that the expected influence centrality is more appropriate for the network that has both positive and negative edges (46). Therefore, the bridge expected influence (i.e., the sum of the edge weights connecting a given node to all nodes in the opposite community) was calculated to quantify the relative importance of individual nodes in explaining cross-community co-occurrences (33). The higher the positive value of bridge expected influence, the greater the activation capacity to other communities; the higher the negative value of bridge expected influence, the greater the deactivation capacity to other communities (33). The aforementioned steps were carried out *via* the R-package *networktools* (33).

Three steps were taken to ensure the accuracy and stability of the present network *via* the R-package *bootnet* (47). First, we bootstrapped (with 2,000 bootstrap samples) the 95% confidence interval of all edges within the network to ensure the accuracy of edge weights. Second, we computed the correlation stability (CS) coefficient of bridge expected influence to ensure the stability of this index. This is achieved through a case-dropping bootstrap approach (with 2,000 bootstrap samples). According to the recommendation, the ideal CS-coefficient is above 0.5 and should not be below 0.25 (47). Third, we conducted bootstrapped difference tests (with 2,000 bootstrap samples) for edge weights and bridge expected influence to examine whether two edge weights or two node bridge expected influence differ significantly from one another.

## Results

### Descriptive statistics

The final sample consisted of 199 doctors (female = 130) and 221 nurses (female = 213) aged 22–50 (mean = 32.74, SD = 5.37) years old. Table 1 shows the demographic characteristics of the participants. Table 2 shows abbreviation, mean scores, and standard deviations for each variable selected in the current network.

**Table 1:** Demographic characteristics of the participants.

**Characteristics**	**Variables**	***N* (%) / Mean (SD)**
Profession	Doctor	199 (47.4)
	Nurse	221 (52.6)
Gender	Female	343 (81.7)
	Male	77 (18.3)
Marriage	Married	304 (72.4)
	Single or divorced	116 (27.6)
Educational background	Undergraduate or less	269 (64.0)
	Postgraduate or more	151 (36.0)
Working years	<=5	135 (32.2)
	6–10	150 (35.6)
	>10	135 (32.2)
Job title	Junior	237 (56.4)
	Middle	163 (38.8)
	Senior	20 (4.8)
Age	18–30	155 (36.9)
	31–40	229 (54.5)
	40–50	34 (8.6)

**Table 2:** Abbreviation, mean scores, and standard deviations for each variable selected in the current network.

**Variables**	**Abbr**	**M**	**SD**
**Mental well-being**			
MW1: I have been feeling optimistic about the future	Optimistic about future	4.19	0.86
MW2: I have been feeling useful	Useful	4.18	0.83
MW3: I have been feeling relaxed	Relaxed	3.71	0.97
MW4: I have been feeling interested in other people	Interested	4.22	0.83
MW5: I have had energy to spare	Energy	3.85	0.86
MW6: I have been dealing with problems well	Dealing with problems	4.08	0.75
MW7: I have been thinking clearly	Thinking clearly	4.14	0.74
MW8: I have been feeling good about myself	Good about myself	3.83	0.87
MW9: I have been feeling close to other people	Close	3.88	0.85
MW10: I have been feeling confident	Confident	3.78	0.91
MW11: I have been able to make up my own mind about things	Make up	4.15	0.76
MW12: I have been feeling loved	Loved	3.96	0.84
MW13: I have been interested in new things	New things	4.09	0.87
MW14: I have been feeling cheerful	Cheerful	3.99	0.89
**Burnout**			
B1: I feel emotionally drained from my work	Emotionally drained	1.59	1.35
B2: I feel used up at the end of the day	Used up	1.80	1.52
B3: I feel tired when I get up in the morning and have to face another day at work	Tired	1.18	1.37
B4: Working with people all day is a real strain for me	Strain	1.23	1.39
B5: I feel burned out from my work	Burned out	0.75	1.15
B6: I have become more callous toward work since I took this job	Callous	0.70	1.10
B7: I have become less enthusiastic about my work	Less enthusiastic	0.81	1.15
B8: I doubt the significance of my work	Doubt significance	0.65	1.03
B9: I have become more and more indifferent in the contribution of my job	Indifferent	0.60	1.06
B10: I deal effectively with the problems of clients^*^	Effectively	1.07	1.22
B11: I feel that I am contributing to my company^*^	Contributing	1.15	1.30
B12: In my opinion, I am good at my job^*^	Good at job	1.05	1.21
B13: I feel very happy when I accomplish some tasks of my job^*^	Happy	0.93	1.22
B14: I have accomplished many worthwhile things in this job^*^	Worthwhile	1.28	1.34
B15: I am confident that I can accomplish all tasks effectively^*^	Accomplish all tasks	1.05	1.25

Abbr, Abbreviation; M, Mean, SD, standard deviation.

^*^Reverse scoring item.

### Network structure

**Figure 1:**
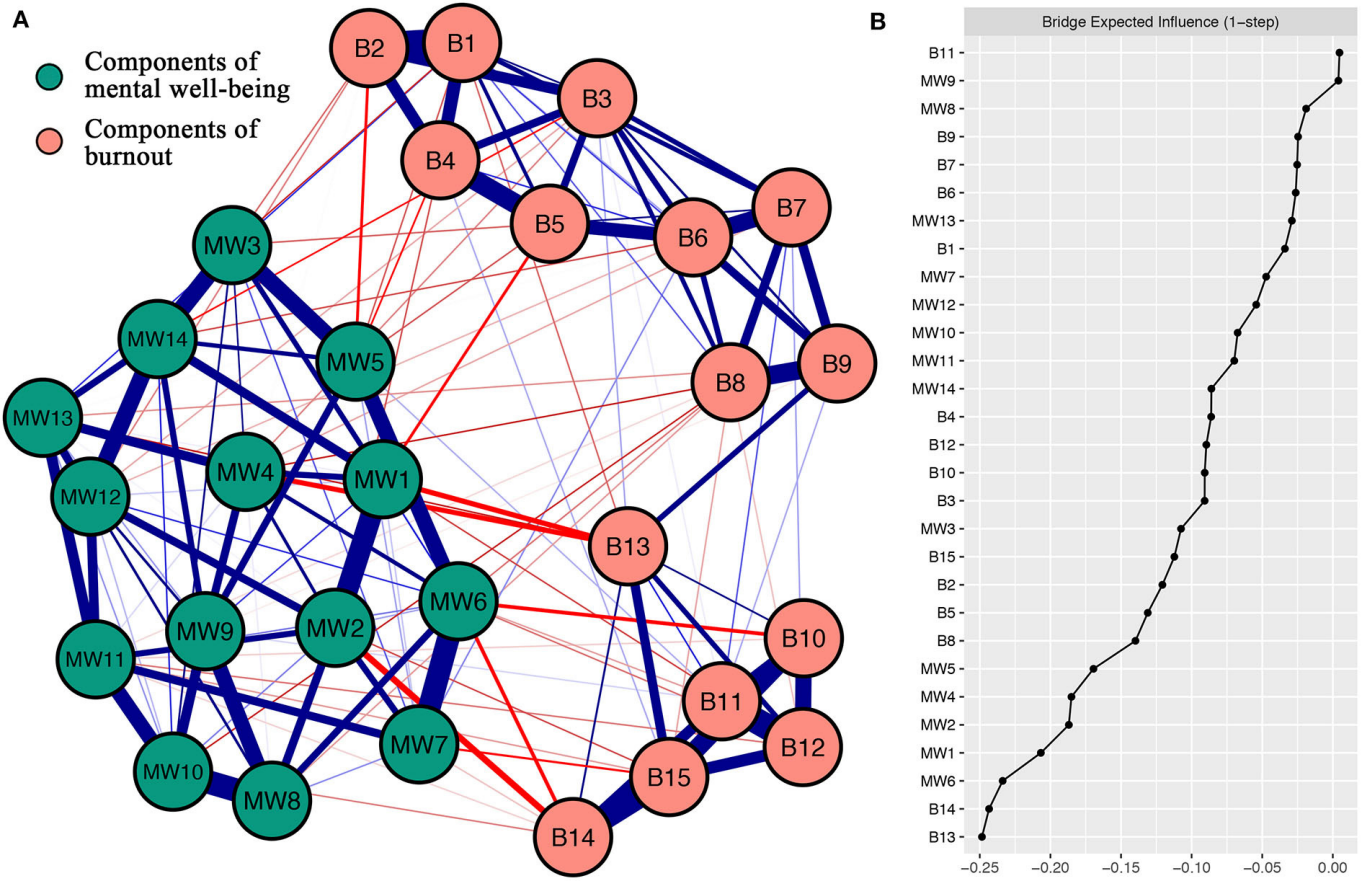
**(A)** Network construction of different components of mental well-being and burnout. Blue edges delegate positive correlations, and red edges delegate negative correlations. The magnitude of the correlation was reflected by the thickness of the edge. Cut value = 0.05. **(B)** Centrality plot drawing the bridge expected influence of per variable selected in the present network (raw score). The text of components of mental well-being and burnout can be seen in Table 1.

The network construction of diverse components of MW and burnout is shown in Figure 1A. There are 47 of 210 (22%) possible edges (weight range from -0.12 to 0.04) within the network. Overall, more negative edges (*n* = 43) were observed than positive edges (*n* = 4). The strongest negative between-community edges were MW2 (“Useful”) – B14 (“Worthwhile”; weight = -0.12), MW1 (“Optimistic about future”) – B13 (“Happy”; weight = -0.10), MW4 (“Interested”) – B13 (“Happy”; weight = -0.10), MW6 (“Dealing with problems”) – B10 (“Effectively”; weight = -0.08), MW6 (“Dealing with problems”) – B14 (“Worthwhile”; weight = -0.08), MW1 (“Optimistic about future”) – B5 (“Burned out”; weight = -0.07), MW5 (“Energy”) – B2 (“Used up”; weight = -0.07), and MW7 (“Thinking clearly”) – B15 (“Accomplish all tasks”; weight = -0.06). In addition, six strongest within-community positive edges have been found in the current network. Such as MW8 (“Good about myself”) – MW10 (“Confident”; weight = 0.37) in the MW community and B1 (“Emotionally drained”) – B2 (“Used up”; weight = 0.47) in the burnout community. The bootstrapped 95% confidence interval is relatively narrow, indicating that edges in the present network are considered to be accurate (Supplementary Figure S1). The bootstrap difference test of edge weight is shown in Supplementary Figure S2.

### Bridge expected influence

Figure 1B shows the node bridge expected influence. In the MW community, MW1 (“Optimistic about future”) and MW6 (“Dealing with problems”) have the lowest bridge expected influence. This displays those in the community of MW have the strongest negative link with burnout components from the network perspective. In the community of burnout, B13 (“Happy”) and B14 (“Worthwhile”) have the lowest bridge expected influences. This shows these two components have the strongest negative connections in the community of burnout with MW components from the network perspective. The CS-coefficient of node bridge expected influence is 0.52, manifesting the centrality index (i.e., bridge expected influence) is sufficiently stable (Supplementary Figure S3). The bootstrapped difference tests for node bridge expected influence have been shown in Supplementary Figure S4.

## Discussion

To the best of our knowledge, this is the first study examining the component-level relationship between MW and burnout among medical staff during the COVID-19 pandemic. These findings may provide new theoretical viewpoints to comprehend the relations between MW and burnout.

Within the MW-burnout network, we found that most between-community edges were negative. It is reasonable that burnout negatively correlates with MW in medical staff during the COVID-19 pandemic (15, 16), which also verifies our first hypothesis. In addition, the strongest between-community edges are existing between positive emotions and functions and professional efficacy (i.e., MW2 “Useful” – B14 “Worthwhile”; MW1 “Optimistic about future” – B13 “Happy”). Unlike our second hypothesis, the strongest negative edges occur between MW and the decreased professional efficacy dimension of burnout in the current study. According to previous studies, the decreased professional efficacy of Chinese medical staff has become a core problem of burnout (48). Medical staff with a high sense of MW experience may respond to various life events in a positive way, resulting in increased job satisfaction and perceived professional efficacy during the COVID-19 pandemic (14, 35). This finding is similar to some studies of burnout in medical staff, which found that negative emotions, impaired interpersonal relationships, and self-denial relate to burnout (49, 50). Specifically, individual medical staff with positive emotions and functions are unlikely to have burnout in the face of work (49, 50). Edge MW2 (“Useful”) – B14 (“Worthwhile”) and MW6 (“Dealing with problems”) – B14 (“Worthwhile”) revealed a link between self-efficacy and professional efficacy, which was similar to previous studies (51). Individuals with high levels of self-efficacy feel more meaningful about their work, which may reduce burnout (51). Edge MW1 (“Optimistic about future”) – B13 (“Happy”) and MW4 (“Interested”) – B13 (“Happy”) demonstrates the association of positive emotions and good interpersonal relationships with professional efficacy. Previous studies have shown that positive emotions and good interpersonal relationships make it easier for individuals to find happiness at work and improve their sense of professional efficacy (52, 53).

Moreover, the final network structure showed that the within-community edges are primarily positive. Within the MW community, three positive edges with the strongest weights were MW8 (“Good about myself”) – MW10 (“Confident”), MW6 (“Dealing with problems”) – MW7 (“Thinking clearly”), and MW1 (“Optimistic about future”) – MW2 (“Useful”). A previous study used network analysis to explore the network structure of MW in four UK cohorts, and also found strong associations between MW8 and MW10 as well as between MW6 and MW7 (29). Take MW1–MW2, for example, optimism about the future may be the embodiment of ability and associated with increased confidence (54, 55). Therefore, when a person is optimistic about his/her future, he/she may find himself/herself useful. Within the burnout community, three positive edges with the highest weights were B1 (“Emotionally drained”) – B2 (“Used up”), B11 (“Contributing”) – B12 (“Good at the job”), and B14 (“Worthwhile”) – B15 (“Accomplish all tasks”). Our previous study used network analysis to examine the network structure of burnout in Chinese nurses, and also found strong associations among these three edges. Take B1 (“Emotionally drained”) – B2 (“Used up”), for example, two items describe the fatigue and lack of enthusiasm caused by work.

Within the current network, node bridge centrality may help to understand the relative importance of each MW component in relation to burnout (56–58). Addressing the bridge node could deactivate the propagation path and reduce co-occurrence (33). Therefore, bridge centrality may provide new insights on the intervention of medical staff's burnout from MW during the COVID-19 pandemic. Within the MW community, MW6 (“Dealing with problems”) and MW1 (“Optimistic about future”) have the lowest value of bridge expected influence, indicating positive emotions (optimistic) and good problem-solving ability of MW were the important nodes bridging the burnout community. The above findings are consistent with our third hypothesis. Thus, targeting these two MW components may be more effective at reducing burnout. Results from intervention-based studies suggested that fostering problem-solving skills and positive emotions may help alleviate burnout (35, 36). Theoretically, optimistic attitudes and problem-solving skills are fundamental for medical staff to maintain their mental health under pandemic-related stress (59, 60). Some targeted interventions for burnout are rooted in cultivating optimism and improving working ability (59, 60). Within the community of burnout, components B13 (“Happy”) and B14 (“Worthwhile”) have the lowest value of bridge expected influence. This indicates that these two components of burnout have stronger negative connections with MW components. Thus, B13 (“Happy”) and B14 (“Worthwhile”) might be more susceptible to the MW community.

This study has some limitations. First, the sample only included medical staff from China, which may limit the generalizability of our conclusion. When examined by the medical staff of other countries, the related network characteristics (such as edges and bridge expected influence) may be different. In addition, the samples in the present study are relatively small. Therefore, although the presented network meets the robustness requirement, conclusions drawn from this sample should be interpreted with caution. Future studies may benefit from adopting a multicenter study design with an increased sample size. Second, the network structure of components of MW and burnout was obtained from cross-sectional data. Thus, no causal relationship can be drawn from current results. Even though previous studies suggested that MW could affect burnout, it does not rule out that burnout may affect MW or they interact with each other. The directed acyclic graph (DAG) is a sophisticated approach for researchers to explore potential causal relationships among nodes in cross-sectional data and generate hypotheses (61, 62). As the current data did not meet the assumption posed by DAG (i.e., measure all confounds) (63–65), we did not apply the DAG approach to generate potential causal hypotheses (62). Future studies that meet the data requirement may consider using DAG to explore potential causal relationships between MW and burnout. Third, this network investigates the network characteristics at the group level. It is possible that these network features of individuals may not be replicated in the similar way. Fourth, other confounding factors, such as demographic characteristics (e.g., working years and working hours), personality, resilience, and empathy (66–69), may also influence the relation between MW and burnout. Therefore, future research could further explore the MW-burnout network under the control of these confounding factors. Last but not least, the current study used network analysis as a bottom-up data-driven approach for exploratory analysis. Thus, future confirmatory studies are required to validate the current findings.

## Conclusion

Notwithstanding the limitations above, this research has important theoretical and clinical value. As far as we know, this research is the first article to study the network structure of MW and burnout in Chinese medical staff during the COVID-19 pandemic. On the one hand, the between-community edges may enrich the potential theoretical mechanism of the relationship between MW and burnout. On the other hand, the bridge expected influence centrality may provide some suggestions (i.e., promoting optimistic attitudes and problem-solving skills) for relevant prevention and intervention to address the requirements of reducing burnout in this special group during the COVID-19 pandemic.

## Data availability statement

The raw data supporting the conclusions of this article will be made available by the authors, without undue reservation.

## Ethics statement

The studies involving human participants were reviewed and approved by Ethics Committee of the First Affiliated Hospital of the Fourth Military Medical University. The patients/participants provided their written informed consent to participate in this study.

## Author contributions

CC, LR, and QY developed the study idea and design. CC, LR, and FL wrote the original draft of this manuscript. All authors contributed to revising subsequent versions of the paper and approved the submitted version.

## Funding

LR's involvement in this research was funded by the Fourth Military Medical University (2021JSTS30).

## Conflict of interest

The authors declare that the research was conducted in the absence of any commercial or financial relationships that could be construed as a potential conflict of interest.

## Publisher's note

All claims expressed in this article are solely those of the authors and do not necessarily represent those of their affiliated organizations, or those of the publisher, the editors and the reviewers. Any product that may be evaluated in this article, or claim that may be made by its manufacturer, is not guaranteed or endorsed by the publisher.
