# Supplementary material for: The relations between mental well-being and burnout in medical staff during the COVID-19 pandemic: A network analysis
Source: Front Public Health. 2022 Aug 10;10:919692. doi: 10.3389/fpubh.2022.919692 (PMC9399609; doi:10.3389/fpubh.2022.919692)
Supplement: Supplementary file 1 [file Data_Sheet_1.DOCX]

**Supplementary Materials**

**The results of MW-burnout network**

1. Figure S1. Accuracy of edge weights
2. Figure S2. Bootstrapped difference test for edge weights
3. Figure S3. Stability of node bridge expected influences
4. Figure S4. Bootstrapped difference test for node bridge expected influences

Figure S1. Accuracy of edge weights

Note: The red line depicts the sample edge weights and the gray bar depicts the bootstrapped confidence interval.

Figure S2. Bootstrapped difference test for edge weights

*Note*: Gray boxes indicate edge weights that do not differ significantly from one another, while black boxes indicate edge weights that do differ significantly. Blue and red boxes on the diagonal correspond to edge weights with positive and negative correlations, respectively.

Figure S3. Stability of node bridge expected influences

*Note*: The red bar represents the average correlation between node bridge expected influences in the full sample and subsample with the red area depicting the 2.5th quantile to the 97.5th quantile.

Figure S4. Bootstrapped difference test for node bridge expected influences

*Note*: Gray boxes indicate node bridge expected influences that do not differ significantly from one another, while black boxes indicate node bridge expected influences that do differ significantly.
